# Determinants of severe QT_c_ prolongation in a real-world gerontopsychiatric setting

**DOI:** 10.3389/fpsyt.2023.1157996

**Published:** 2023-03-23

**Authors:** Martin Schulze Westhoff, Sebastian Schröder, Johannes Heck, Tabea Pfister, Kirsten Jahn, Olaf Krause, Felix Wedegärtner, Stefan Bleich, Kai G. Kahl, Tillmann H. C. Krüger, Adrian Groh

**Affiliations:** ^1^Department of Psychiatry, Social Psychiatry and Psychotherapy, Hannover Medical School, Hannover, Germany; ^2^Hannover Medical School, Institute for Clinical Pharmacology, Hannover, Germany; ^3^Hannover Medical School, Institute for General Practice and Palliative Care, Hannover, Germany; ^4^Center for Medicine of the Elderly, DIAKOVERE Henriettenstift, Hannover, Germany; ^5^Center for Systemic Neursocience, Hannover, Germany

**Keywords:** QT_c_ prolongation, geriatrics, geriatric psychiatry, drug safety, AzCERT classification, elderly

## Abstract

**Introduction:**

QT_c_ prolongation carries the risk of ventricular tachyarrhythmia (Torsades de Pointes) and sudden cardiac death. Psychotropic drugs can affect ventricular repolarization and thus prolong the QT_c_ interval. The present study sought to investigate the risk factors (pharmacological and non-pharmacological) of severe QT_c_ prolongation in gerontopsychiatric patients.

**Methods:**

Electrocardiograms of patients on a gerontopsychiatric ward were screened for QT_c_ prolongation. Medication lists were examined utilizing the AzCERT classification. Potential drug interactions were identified with the electronic drug interaction program mediQ.

**Results:**

The overall prevalence of QT_c_ prolongation was 13.6%, with 1.9% displaying severe QT_c_ prolongation (≥ 500 ms). No statistically significant differences between patients with moderate and severe QT_c_ prolongation were identified; however, patients with severe QT_c_ prolongation tended to take more drugs (*p* = 0.063). 92.7% of patients with QT_c_ prolongation took at least one AzCERT-listed drug, most frequently risperidone and pantoprazole. Risperidone and pantoprazole, along with pipamperone, were also most frequently involved in potential drug interactions. All patients displayed additional risk factors for QT_c_ prolongation, particularly cardiac diseases.

**Conclusion:**

In addition to the use of potentially QT_c_-prolonging drugs, other risk factors, especially cardiac diseases, appear to be relevant for the development of QT_c_ prolongation in gerontopsychiatric patients. Pantoprazole was frequently involved in potential drug interactions and should generally not be used for more than 8 weeks in geriatric populations. As clinical consequences of QT_c_ prolongation were rare, potentially QT_c_-prolonging drugs should not be used overcautiously; their therapeutic benefit should be considered as well. It is paramount to perform diligent benefit–risk analyses prior to the initiation of potentially QT_c_-prolonging drugs and to closely monitor their clinical (side) effects.

## Introduction

The QT interval in the electrocardiogram (ECG) comprises the time from the beginning of the QRS complex to the end of the T wave and reflects ventricular repolarization (1). The QT interval depends on the heart rate; therefore, various formulas (e.g., according to Bazett, Hegglin, Fridericia, and Framingham) have been developed to calculate the rate-corrected QT (QT_c_) interval (2). A prolonged QT_c_ interval in the ECG indicates impaired ventricular repolarization and is associated with the occurrence of certain ventricular tachyarrhythmias, so-called torsades de pointes (TdP), and sudden cardiac death (SCD) (3). A prolonged QT_c_ interval is considered to start at 450 ms in men and 470 ms in women (4). Above 500 ms, the probability of occurrence of TdP and SCD is significantly increased across genders (3, 4). Pathophysiologically, a prolonged QT_c_ interval is elicited by a dysfunction of certain cardiac sodium or potassium channels, either congenital (due to specific gene mutations) or acquired. Acquired forms of QT_c_ prolongation occur more frequently than congenital forms (5). A common reason for acquired QT_c_ prolongation is the intake of certain drugs that interact with cardiac ion channels and may thus lead to disturbances in ventricular repolarization (6). Paradoxically, this applies in particular to the class of antiarrhythmic drugs, but also to certain antibiotics (e.g., macrolide antibiotics) and many psychotropic drugs (6, 7). However, there exist numerous other risk factors for prolongation of the QT_c_ interval, such as cardiac diseases, thyroid dysfunction, electrolyte disturbances (e.g., hypokalemia, hyponatremia), or age > 65 years (8, 9).

The investigation of at-risk populations for QT_c_ prolongation is of paramount importance (10, 11). Gerontopsychiatric patients represent a high-risk population due to their age, presence of somatic comorbidities, and frequent use of psychotropic drugs that potentially extend the QT_c_ interval (10, 12). Due to altered pharmacodynamic and pharmacokinetic properties, along with frequent polypharmacy, the probability of occurrence of adverse drug reactions (ADRs) is significantly increased in geriatric patients (13, 14). In clinical practice, prolongations of the QT_c_ interval are often suspected to be caused by psychotropic drugs without considering the presence of other risk factors (15). This, in turn, can lead to potential drug prescribing omissions (PPOs), if clinically indicated drugs are withheld due to fears of QT_c_ prolongation. PPOs carry the risk of worsening psychopathology (16, 17).

The aim of our study was to investigate the determinants and risk factors of severe compared to moderate QT_c_ prolongation in gerontopsychiatric patients. For this purpose, the ECGs of patients on a gerontopsychiatric ward of a large university hospital in Germany were screened for prolonged QT_c_ intervals. Using the Arizona Center for Education and Research on Therapeutics (AzCERT) classification of potentially QT_c_-prolonging drugs, patients’ medication lists were analyzed (18). In addition, drug interactions with potential impact on ventricular repolarization were explored.

## Methods

### Ethics approval

This study was approved by the Ethics Committee of Hannover Medical School (No. 10595_BO_K_2022) and adheres to the Declaration of Helsinki (1964) and its later amendments (current version from 2013).

### Eligibility criteria

Patients were enrolled in the study (i) if they were ≥ 65 years of age, (ii) if they were treated on the gerontopsychiatric ward of the Department of Psychiatry, Social Psychiatry and Psychotherapy of Hannover Medical School between 01 January 2014 and 31 December 2021, (iii) if they or their legal representative had provided written informed consent that patient-related data be used for clinical research, and (iv) if they exhibited a QT_c_ prolongation in the ECG (for definition see next paragraph), which was confirmed by manual ECG re-evaluation.

Hannover Medical School is a large university hospital and tertiary care referral center in northern Germany. The gerontopsychiatric ward is a 27-bed facility specialized on the treatment and care of elderly psychiatric inpatients.

### Categorization of QTc prolongation

The length of the QT_c_ interval was calculated with Bazett’s formula. According to the criteria of the European Medicines Agency (EMA), QT_c_ intervals ≥ 450 ms in men and ≥ 470 ms in women were categorized as prolonged (19). Moderate QT_c_ prolongation was defined as a prolonged QT_c_ interval < 500 ms. Severe QT_c_ prolongation was defined as a QT_c_ interval ≥ 500 ms (19). 12-lead ECG machines were used in our study, whereby ECGs were scanned into.pdf formats. In a first step, all patients with a prolonged QT_c_ interval in the automatic electronic calculation of ECG parameters were identified. In the next step, the ECGs of these patients were manually re-evaluated. To this end, manual calipers were used and the tangent method was applied to determine the end of the T wave. The length of the QT_c_ interval was determined in lead II. RR and QT_c_ intervals were averaged across several beats. U waves as correlates of late repolarization were assessed in leads V_2_ and V_3_, and—if present—were not included in the calculation of QT_c_ intervals. In patients with a heart rate > 100 beats per minute (bpm), the QT_c_ interval was calculated with Fridericia’s formula (1). In the presence of right and/or left bundle branch blocks, Bogossian’s formula was used to calculate the QT_c_ interval (1, 20). ECGs with numerous artifacts and flat T waves were excluded.

### Medication chart reviews, drug interaction checks, risk factors for QT_c_ prolongation, and demographic characteristics

Medication charts of enrolled patients were analyzed by an interdisciplinary team of experts in psychiatry, internal medicine, and clinical pharmacology. Regularly taken drugs were assessed with the aid of the AzCERT classification (9, 18).

AzCERT is part of the Critical Path Institute established by the United States Food and Drug Administration (FDA) and is one of 14 centers dedicated to improving drug development processes (18). AzCERT maintains CredibleMeds, an online database which categorizes the risk of individual drugs to prolong the QT_c_ interval and/or to elicit TdP (18). Three main categories are differentiated:

1. Drugs that, under normal clinical conditions, significantly increase the risk for QT_c_ prolongation/TdP (“known risk”).
2. Drugs with known capacity to prolong the QT_c_ interval but with lacking evidence regarding the development of TdP (“possible risk”).
3. Drugs with a conditional risk for QT_c_ prolongation/TdP when given in excessive dosages or in the presence of other risk conditions (“conditional risk”).

Drug interaction checks were performed with mediQ (Psychiatrische Dienste Aargau AG, mediQ Kompetenzzentrum für Medikamentensicherheit, Windisch, Switzerland), an electronic drug interaction program specialized on psychopharmaceuticals. mediQ categorizes the clinical severity of drug interactions as “low,” “average,” or “high.” For the purpose of our study, drug interactions with an association to possible QT_c_ prolongation were considered. Thus, for each patient case, potential interaction pairs and the AzCERT categories of the involved drugs were recorded.

Demographic characteristics—i.e., age, sex, and International Statistical Classification of Diseases and Related Health Problems 10th Revision (ICD-10) diagnoses—were retrieved from patient records. We used the Chronic Kidney Disease Epidemiology Collaboration (CKD-EPI) formula to calculate estimated glomerular filtration rates (eGFR). Hospital discharge letters were used to identify cases in which an acute cardiac event occurred during the hospital stay and cases in which the medication was changed due to QT_c_ prolongation.

### Statistical analysis

All statistical analyses were conducted with IBM SPSS Statistics for Windows, version 28 (Armonk, New York, NY, United States). Descriptive statistical methods were used to summarize the data. Quantitative variables were tested for normal distribution with the Shapiro–Wilk test and by inspection of the histogram and Q–Q plot. Due to skewed distribution, quantitative variables are depicted as medians with interquartile ranges (IQRs). For quantitative variables, differences between patients with moderate and patients with severe QT_c_ prolongation were analyzed with the Mann–Whitney *U* test for independent samples. Categorical variables are displayed as absolute and relative frequencies. For categorical variables, differences between patients with moderate and patients with severe QT_c_ prolongation were analyzed with Pearson’s chi-squared test or Fisher’s exact test. Fisher’s exact test was preferred if any of the four cells of a 2 × 2 table had less than five observations. *p* values < 0.05 were considered statistically significant. Due to the exploratory nature of our investigation, no adjustments for multiple testing were made.

## Results

### Study population

**Figure 1:**
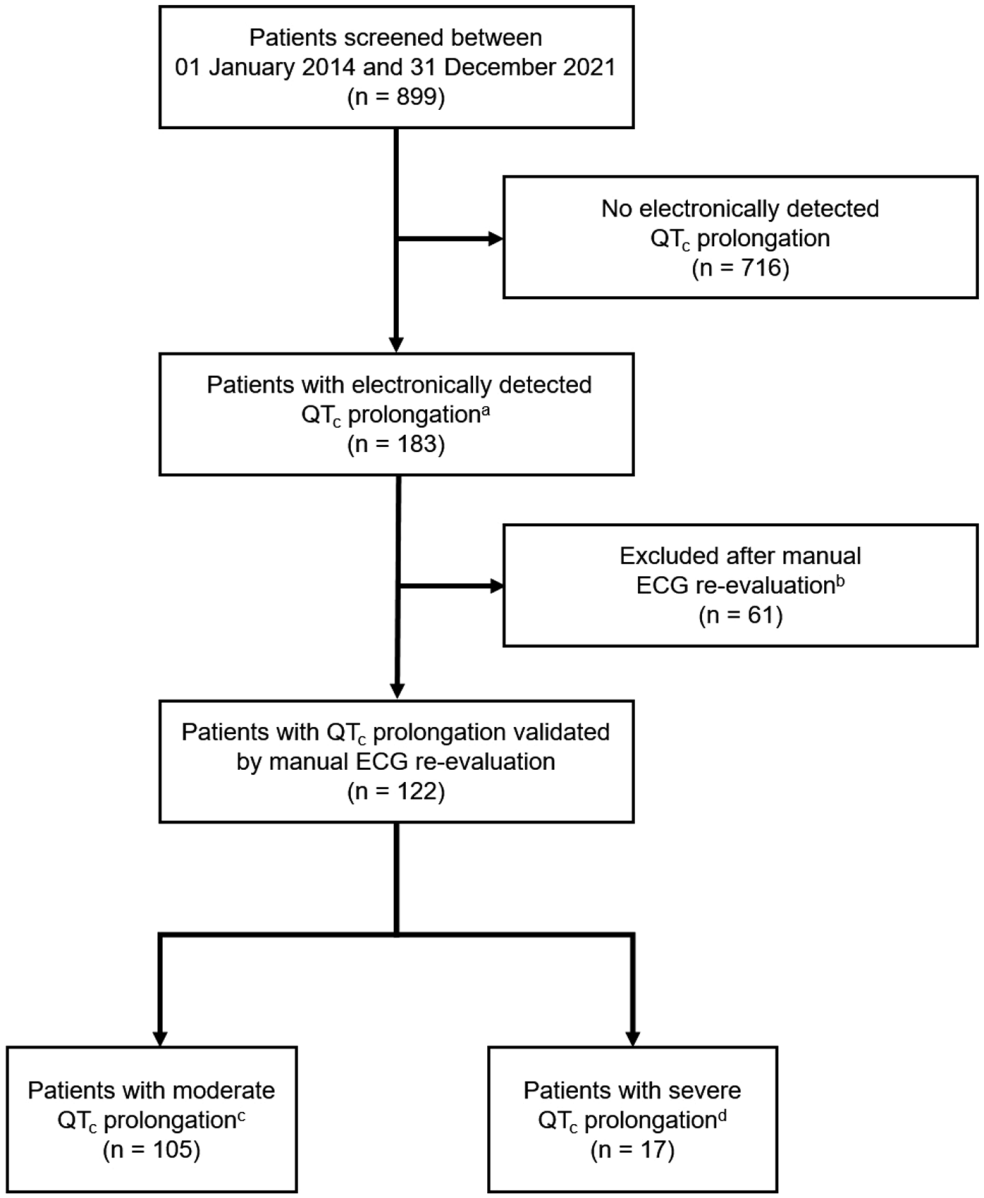
Flow of patients. ^a^QTc intervals ≥ 450 ms in men/≥ 470 ms in women were considered prolonged. ^b^E.g., due to artifacts. ^c^Moderate QTc prolongation was defined as a prolonged QTc interval < 500 ms. ^d^Severe QTc prolongation was defined as a QTc interval ≥ 500 ms (irrespective of gender). ECG, electrocardiogram; QT_c_, rate-corrected QT.

One hundred and twenty-two of 899 screened patients (13.6%) fulfilled the eligibility criteria and were enrolled in the study (Figure 1). The median age of the study population (*n* = 122) was 77 years (IQR 70–83 years; minimum 65 years; maximum 99 years) and 38.5% (47/122) of the patients were female (Table 1). The median QT_c_ interval duration in the study population was 477 ms (IQR 466–490 ms; minimum 451 ms; maximum 525 ms). 86.1% (105/122) of the patients displayed a moderate QT_c_ prolongation, while 13.9% (17/122) exhibited a severe QT_c_ prolongation. The median eGFR in the study population was 67 ml/min (IQR 49–81.25 ml/min; minimum 15 ml/min; maximum 103 ml/min). The patients took a median of 7.5 drugs (IQR 4–9 drugs; minimum 0 drugs; maximum 18 drugs), with a median of 2 AzCERT-listed drugs (IQR 1–3 AzCERT-listed drugs; minimum 0 AzCERT-listed drugs; maximum 6 AzCERT-listed drugs). Dementia was the most frequent psychiatric diagnosis in the study population (40.2%; 49/122). The most prevalent somatic disorder was arterial hypertension, which affected 77.9% (95/122) of the patients. Other frequent risk factors for QT_c_ prolongation in our study population were chronic heart failure (41.0%; 50/122) and coronary heart disease (32.8%; 40/122).

**Table 1:** Characteristics of the study population (*n* = 122).

Variables	*n*	%
*Sex*		
Female	47	38.5
Male	75	61.5
*Psychiatric diagnoses* *^a^*		
Depression^b^	27	22.1
Bipolar affective disorder^c^	12	9.8
Schizophrenia or schizophreniform disorder^d^	22	18.0
Mental and behavioral disorder due to use of alcohol, tobacco, or sedatives or hypnotics^e^	28	23.0
Dementia^f^	49	40.2
Delirium^g^	27	22.1
Other psychiatric disorder(s)	15	12.3
*Somatic diagnoses* *^a^*		
Arterial hypertension	95	77.9
Coronary heart disease	40	32.8
Chronic heart failure	50	41.0
Atrial fibrillation	23	18.9
Cardiac arrhythmia other than atrial fibrillation	40	32.8
Status post stroke	20	16.4
Dyslipidemia	33	27.0
Type-2 diabetes mellitus	24	19.7
Chronic obstructive pulmonary disease	9	7.4
Thyroid dysfunction	34	27.9
Urinary tract infection	19	15.6
Hypokalemia	26	21.3
Hyponatremia	13	10.7
Hypocalcemia	8	6.6
Other somatic disorder(s)	120	98.4

a Patients could have more than one diagnosis;

b ICD-10 F32, F33;

c ICD-10 F31;

d ICD-10 F06.2, F2X;

e ICD-10 F10, F13, F17;

f ICD-10 F00, F01, F02, F03;

g ICD-10 F05.

The median age of the study population was 77 years (interquartile range 70–83 years).

ICD-10, International Statistical Classification of Diseases and Related Health Problems 10th Revision.

### Treatment modifications and cardiac events

The medication was changed in 10.7% (13/122) of patients as a consequence of QT_c_ prolongation. In 23.1% (3/13) of treatment modifications, antidepressants were discontinued, while in 69.2% (9/13) antipsychotic medications were stopped; one case regarded a discontinuation of pantoprazole. Four patients experienced a cardiac event during their hospital stay (myocardial infarction, *n* = 2; malign cardiac arrhythmia, *n* = 2). In one of these cases (one case of malign cardiac arrhythmia), a causal involvement of QT_c_ prolongation was suspected. The respective patient developed a TdP tachyarrhythmia, and also had a severely prolonged QT_c_ interval, as well as various risk factors for TdP (arterial hypertension, chronic heart failure, hyponatremia, and hypokalemia). In the remaining three cases (two cases of myocardial infarction, one case of malign cardiac arrhythmia), a causal involvement of QT_c_ prolongation could not be ruled out. Three of these patients died, one patient recovered with sequelae.

### Comparison between patients with moderate and severe QT_c_ prolongation

There were no statistically significant differences between patients with moderate and patients with severe QT_c_ prolongation regarding renal function or presence of comorbidities previously characterized as risk factors for QT_c_ prolongation (Table 2). We observed non-significant trends towards a higher proportion of females among patients with severe QT_c_ prolongation compared to patients with moderate QT_c_ prolongation [58.8% (10/17) vs. 35.2% (37/105); *p* = 0.064] and towards a higher number of drugs taken [9 drugs (IQR 6–11 drugs) vs. 7 drugs (IQR 4–9 drugs); *p* = 0.063].

**Table 2:** Comparison of patients with moderate and severe QT_c_ prolongation.

Characteristic and category	Total	Patients with moderate QT_c_ prolongation	Patients with severe QT_c_ prolongation	*p* value
	(*n* = 122)	(*n* = 105; 86.1%)	(*n* = 17; 13.9%)	
Median age (IQR)—years	77 (70–83)	77 (70–82.5)	78 (69–84.5)	0.915^a^
Age > 80 years—% (no.)	23.8 (29)	22.9 (24)	29.4 (5)	0.559^b^
Female sex—% (no.)	38.5 (47)	35.2 (37)	58.8 (10)	0.064^b^
Median number of drugs (IQR)	7.5 (4–9)	7 (4–9)	9 (6–11)	0.063^a^
Median number of AzCERT-listed drugs (IQR)	2 (1–3)	2 (1–3)	2 (2–3.5)	0.241^a^
Median eGFR (IQR)—ml/min	67 (49–81.25)	67 (52–80.5)	54 (42–86)	0.464^a^
Arterial hypertension—% (no.)	77.9 (95)	77.1 (81)	82.4 (14)	0.761^c^
Coronary heart disease—% (no.)	32.8 (40)	32.4 (34)	35.3 (6)	0.812^b^
Chronic heart failure—% (no.)	41.0 (50)	39.0 (41)	52.9 (9)	0.280^b^
Type-2 diabetes mellitus—% (no.)	19.7 (24)	20.0 (21)	17.6 (3)	1.000^c^
Dyslipidemia—% (no.)	27.0 (33)	25.7 (27)	35.3 (6)	0.394^c^
Atrial fibrillation—% (no.)	18.9 (23)	19.0 (20)	17.6 (3)	1.000^c^
Cardiac arrhythmia other than atrial fibrillation—% (no.)	32.8 (40)	30.5 (32)	47.1 (8)	0.177^b^
Thyroid dysfunction—% (no.)	27.9 (34)	27.6 (29)	29.4 (5)	1.000^c^
Hypokalemia—% (no.)	21.3 (26)	20.0 (21)	29.4 (5)	0.357^c^
Hyponatremia—% (no.)	10.7 (13)	10.5 (11)	11.8 (2)	1.000^c^
Hypocalcemia—% (no.)	6.6 (8)	5.7 (6)	11.8 (2)	0.309^c^

Moderate QT_c_ prolongation was defined as 450 ms (men)/470 ms (women) ≤ QT_c_ interval < 500 ms. Severe QT_c_ interval prolongation was defined as QT_c_ interval ≥ 500 ms (irrespective of gender).

a Mann–Whitney *U* test for independent samples.

b Pearson’s Chi-squared test.

c Fisher’s exact test.

AzCERT, Arizona Center for Education and Research on Therapeutics; eGFR, estimated glomerular filtration rate; IQR, interquartile range; no., number; QT_c_, rate-corrected QT.

### Characteristics of drug prescriptions and categorization according to the AzCERT classification

In total, 857 medications were prescribed in the study population. The most frequently prescribed drugs were ramipril (4.8%; 41/857) and pantoprazole (4.6%; 39/857), followed by risperidone and lorazepam (each 4.2%; 36/857; Supplementary Table 1). 33.8% (290/857) of the prescribed drugs were indexed on the AzCERT list. 23.0% (28/122) of the patients received one AzCERT-listed drug, while 69.7% took more than one AzCERT-listed drug. 2.3% (20/857) of all prescribed drugs had a known risk of QT_c_ prolongation according to the AzCERT classification, 20.7% (177/857) had a possible risk, and 9.9% (85/857) had a conditional risk. Haloperidol (0.9%; 8/857) and citalopram (0.5%; 4/857) were the most frequently prescribed drugs with a known risk of QT_c_ prolongation. Pipamperone (3.4%; 29/857) and mirtazapine (2.0%; 17/857) were the leading drugs with a possible risk of QT_c_ prolongation, whereas pantoprazole (4.6%; 39/857) and risperidone (4.2%; 36/857) were the most frequently prescribed drugs with a conditional risk of QT_c_ prolongation.

### Drug interaction checks

The drug interactions (266) with an association to possible QT_c_ prolongation were detected in the study population. Overall, potentially QT_c_-prolonging drug interactions were present in 64.8% (79/122) of patients. The most frequent interaction pairs were pipamperone + risperidone (3.8%; 10/266), risperidone + torasemide (3.8%; 10/266), and pantoprazole + risperidone (3.4%; 9/266; Supplementary Table 2). The interaction potential of the two pairs risperidone + citalopram and amiodarone + tramadol (0.8%; 2/266) was categorized as “high” by mediQ. 45.1% (120/266) of the interaction pairs were considered to have an “average” interaction potential, while 54.1% (144/266) exhibited a “low” interaction potential. In the case of the interaction pair chlorprothixene + haloperidol, both involved drugs had a known risk of QT_c_ prolongation according to the AzCERT classification. Risperidone (25.2%; 67/266), pipamperone (19.5%; 52/266), pantoprazole (15.4%; 41/266), and quetiapine (15.4%; 41/266) were most frequently involved in drug interactions. 10.9% (29/266) and 89.1% (237/266) of the interactions were characterized as primarily pharmacokinetic and primarily pharmacodynamic, respectively. The most frequent pharmacokinetic interaction pair was melperone + risperidone (2.6%; 7/266; increased plasma concentration of risperidone due to inhibition of CYP2D6 by melperone).

## Discussion

The present study investigated the frequency and risk determinants of severe compared to moderate QT_c_ prolongation in a gerontopsychiatric patient population in the setting of a large university hospital in Germany. Emphasis was put on investigating prescription characteristics of drugs with potential QT_c_-prolonging effects according to the AzCERT classification (18). Furthermore, the number and severity of drug interactions with association to potential QT_c_ prolongation were analyzed.

In psychiatric patients, the frequency of QT_c_ prolongation was investigated in several studies, with heterogeneous results (21–23). The prevalence of an at least moderate QT_c_ prolongation ranged from 1 to 10% of patients (21–23), while the proportion of patients with severe QT_c_ prolongation (> 500 ms) varied between 0.2 and 3% (21, 24, 25). Different study designs, enrollment of both inpatients and outpatients, as well as different age profiles of the participants may serve as explanations for these discrepancies. To date, three studies investigated the characteristics of QT_c_ prolongation in geriatric psychiatry (26–28). Dumontet et al. (28) found that in a sample of 88 inpatients, 29.4% of men and 21.4% of women displayed QT_c_ prolongation. In a more recent study from India by Das et al. (26), the prevalence of QT_c_ prolongation was reported to be 29.4%, with 1.8% of all study participants exhibiting a QT_c_ interval of > 500 ms. These data referred to patients in gerontopsychiatric outpatient care (26). A previous study by Das et al. (27) with a smaller sample size estimated the prevalence of QT_c_ prolongation to be 19.2% in men and 10.3% in women. In our study, the prevalence of QT_c_ prolongation tended to be lower (13.6%); however, the proportion of severe QT_c_ prolongation (1.9%) was comparable to the recent Das et al. (26) study.

The higher prevalence of QT_c_ prolongation in gerontopsychiatric patients compared with general psychiatric settings can be explained by the advanced age of patients and age-associated multimorbidity. The markedly lower proportion of QT_c_ prolongation in our study, in turn, may be due to more narrowly defined inclusion criteria. For example, we did not solely rely on automatic calculations of ECG parameters. Instead, all ECGs suspicious of QT_c_ prolongation were re-examined manually, taking influences of heart rate and bundle branch blocks into consideration. This led to the exclusion of one-third (61/183) of automatically detected QT_c_ prolongations.

The most common psychiatric diagnoses in our study population were dementia, substance use disorders, depression, and delirium, which is comparable to other studies (26, 29, 30). Previous studies examined the frequency and significance of risk factors for QT_c_ prolongation in psychiatric patients (21, 23, 24, 31). In this regard, the influence of potentially QT_c_-prolonging drugs has been emphasized (26, 32). A study from Pakistan found that 91.6% of psychiatric inpatients were taking potentially QT_c_-prolonging drugs, which was the most common risk factor (32). These and other results have led to a sometimes overcautious prescription of potentially QT_c_-prolonging drugs in clinical practice, which may represent a PPO under certain circumstances (15, 17). In fact, studies on the effect of medication on QT_c_ prolongation in the psychiatric context had varying results (22, 33). Results were also heterogeneous in gerontopsychiatric patient groups (26, 28). Whereas in the Das et al. study all patients with QT_c_ prolongation received potentially QT_c_-prolonging drugs, Dumontet et al. (26, 28) found that 57.9% of patients with QT_c_ prolongation were not taking QT_c_-prolonging drugs. Risk factors especially for severe QT_c_ prolongation have not been investigated to date.

In the present study, 92.7% of patients with QT_c_ prolongation received at least one potentially QT_c_-prolonging drug according to the AzCERT classification. In addition to age, which was set at ≥ 65 years as part of the inclusion criteria and which represents an independent risk factor for QT_c_ prolongation, the prescription of AzCERT-listed drugs was the most frequent risk factor in our study population. Of note, at least one additional risk factor was identified in all patients, most notably cardiac diseases such as arterial hypertension and chronic heart failure, which affected 77.9 and 51% of patients, respectively. In general, patients in our study population displayed higher proportions of risk factors, especially cardiac diseases, than in the previous studies in the gerontopsychiatric setting. This may be explained by the fact that our investigation focused exclusively on patients with prolonged QT_c_ intervals, but also emphasizes that not only QT_c_-prolonging drugs but presumably a combination of different risk factors seems to be responsible for the development of QT_c_ prolongation. We did not observe statistically significant differences between patients with moderate and severe QT_c_ prolongation; however, there was a trend towards a higher number of drugs taken in the group with severe QT_c_ prolongation. This opens avenues for follow-up studies with a prospective design, which should investigate whether polypharmacy represents a risk factor for severe QT_c_ prolongation.

Four patients (3.3% of all patients with QT_c_ prolongation) developed a cardiac event during their hospital stay, a proportion that was somewhat higher than in previous investigations (26, 32, 33). Yet, a causal relation to QT_c_ prolongation was suspected in only one of these four cases.

To the best of our knowledge, our study is the first to investigate the frequency of treatment modifications as a consequence of QT_c_ prolongation in geriatric psychiatry. Although 92.7% of the patients took at least one AzCERT-listed drug, the medication was changed in only 10.7% of cases as a consequence of QT_c_ prolongation, with antipsychotic drugs being discontinued in 69.2% of treatment modifications. This suggests that QT_c_ prolongations were often tolerated in clinical routine in view of the patients’ high-risk profiles for QT_c_ prolongation, and were less frequently causally attributed to the influence of medication. Nevertheless, antipsychotics in particular appeared to be often associated with QT_c_ prolongation by the treating physicians.

In the present study, the majority of patients (69.7%) were taking more than one AzCERT-listed drug, which is comparable with findings by Das et al. (26). In our study, the largest proportion of AzCERT-listed drugs were those with a possible risk for QT_c_ prolongation. These accounted for 20.7% of all drugs, which was markedly higher than the proportions of the other AzCERT categories. For example, drugs with a known risk for QT_c_ prolongation only accounted for 2.3%. In previous studies, the proportion of drugs with a known risk for QT_c_ prolongation was considerably higher than in our investigation (26, 33, 34).

The most frequently prescribed drugs with a known risk for QT_c_ prolongation in our study were haloperidol and citalopram. In previous studies in the psychiatric setting, these drugs were also among the most frequently prescribed substances in this category, along with levomepromazine and chlorpromazine (26, 32, 34). Pipamperone and mirtazapine were the most common drugs with a conditional risk in our study, whereas in other investigations these two drugs were prescribed infrequently, in contrast to lithium and aripiprazole, which were leaders in this category in previous reports (26, 32, 34). Moreover, the most frequently prescribed QT_c_-prolonging drugs in our study were pantoprazole and risperidone, both of which convey a possible risk of QT_c_ prolongation according to the AzCERT classification. Other studies identified quetiapine and sertraline as the most frequently prescribed drugs in psychiatric patients in this category (26, 33, 34).

Das et al. and Hefner et al. (26, 35) investigated the characteristics of drug interactions associated with QT_c_ prolongation in psychiatric patients and identified pipamperone + risperidone and escitalopram + risperidone as the most frequent interaction pairs. Similarly, pipamperone + risperidone represented the most frequent combination with drug interaction potential in terms of QT_c_ prolongation in our study, along with risperidone + torasemide. In addition to risperidone and pipamperone, quetiapine and pantoprazole were also frequently involved in potential interactions, suggesting a significant contribution of these drugs to QT_c_ prolongation.

Of note, pantoprazole was frequently involved in drug interactions associated with potential QT_c_ prolongation. Pantoprazole has been reported to increase QT_c_ and has therefore been added to the list of “Drugs to be avoided in patients with congenital long QT syndrome” (36). Extended use (> 14 days) of proton pump inhibitors (PPIs) should be discouraged because of their inherent risk of TdP (37).

Our investigation is not without limitations. It was designed as a retrospective and unicenter analysis. Similarly, we did not evaluate the evolution of the QT_c_ interval during the course of treatment and did not include the duration of drug intake. It should also be mentioned critically that we focused exclusively on patients with QT_c_ prolongation in our statistical analyses, which led to a relatively small sample size with limited statistical power of the results. The results of our study need to be validated in future studies with a prospective and multicenter design and with larger sample sizes to allow for better generalizability.

In summary, the present study investigated the frequency and characteristics of severe compared to moderate QT_c_ prolongation in geriatric psychiatry. It was striking that almost all patients also suffered from cardiac diseases and displayed other risk factors for QT_c_ prolongation, suggesting a multifactorial genesis of QT_c_ prolongation. Nevertheless, in individual cases, drugs may exert a decisive impact on the QT_c_ interval and potentially result in life-threatening consequences such as TdP. In particular, combinations of drugs with a known risk for QT_c_ prolongation (according to the AzCERT classification) should be re-evaluated critically. On the other hand, fears of QT_c_ prolongation should not result in PPOs.

Strengths of our study were the high quality of ECG assessments (in contrast to previous studies) with stringent exclusion criteria, and analysis of potential differences between patients with moderate and severe QT_c_ prolongation. The results of our work indicate that polypharmacy might be a potential risk factor for severe QT_c_ prolongation, even though this needs to be verified in future studies. Furthermore, our investigation is the first to analyze therapeutic consequences of QT_c_ prolongation. We were able to show that QT_c_ prolongations are often tolerated by the treating physicians, suggesting that the therapeutic benefits of potentially QT_c_-prolonging drugs frequently outweigh their risks in clinical practice. An additional advantage of our study was the comprehensive evaluation of drug interaction pairs which contributed to QT_c_ prolongations in a real-world setting.

## Data availability statement

The original contributions presented in the study are included in the article/Supplementary material, further inquiries can be directed to the corresponding author.

## Ethics statement

The studies involving human participants were reviewed and approved by Ethics Committee of Hannover Medical School. The patients/participants provided their written informed consent to participate in this study.

## Author contributions

MSW and AG: conceptualized the study. MSW, AG, and JH: analyzed the data. JH: inferential statistics, language editing, provided expert advice in clinical pharmacology. MSW, AG, SS, TP, SB, KK, TK, KJ, and FW: provided expert advice in psychiatry and psychopharmacology. OK: provided expert advice in cardiology. MSW, AG, JH, and SS: interpreted the study results, drafted the first version of the manuscript, created the tables and figures. TP, KK, SB, TK, OK, KJ, and FW: assisted with the preparation of the manuscript. AG: supervised the project. All authors contributed to the article and approved the submitted version.

## Conflict of interest

The authors declare that the research was conducted in the absence of any commercial or financial relationships that could be construed as a potential conflict of interest.

## Publisher’s note

All claims expressed in this article are solely those of the authors and do not necessarily represent those of their affiliated organizations, or those of the publisher, the editors and the reviewers. Any product that may be evaluated in this article, or claim that may be made by its manufacturer, is not guaranteed or endorsed by the publisher.
