# Supplementary material for: Determinants of severe QTc prolongation in a real-world gerontopsychiatric setting
Source: Front Psychiatry. 2023 Mar 23;14:1157996. doi: 10.3389/fpsyt.2023.1157996 (PMC10076587; doi:10.3389/fpsyt.2023.1157996)
Supplement: Supplementary file 1 [file Table_1.DOCX]

Supplementary Material

**Determinants of QT_c_ prolongation in a real-world gerontopsychiatric setting**

**Martin Schulze Westhoff^1^, Sebastian Schröder^1^, Johannes Heck^2^, Tabea Pfister^1^, Kirsten Jahn^1^, Olaf Krause^3,4^, Felix Wedegärtner^1^, Stefan Bleich^1^, Kai G. Kahl^1^, Tillmann H. C. Krüger^1^, and Adrian Groh^1^**

^1^Department of Psychiatry, Social Psychiatry and Psychotherapy, Hannover Medical School, Hannover, Germany

^2^Institute for Clinical Pharmacology, Hannover Medical School, Hannover, Germany

^3^Institute for General Practice and Palliative Care, Hannover Medical School, Hannover, Germany

^4^Center for Medicine of the Elderly, DIAKOVERE Henriettenstift, Hannover, Germany

*** Correspondence:**Dr. Martin Schulze Westhoff, MD

Department of Psychiatry, Social Psychiatry and Psychotherapy

Hannover Medical School

Carl-Neuberg-Str. 1

30625 Hannover, Germany

Tel.: +49 511 532-7037

Fax: +49 511 532-18523

E-mail: [schulzewesthoff.martin@mh-hannover.de](mailto:schulzewesthoff.martin@mh-hannover.de)

# Supplementary Tables

**SUPPLEMENTARY TABLE 1** Absolute and relative frequencies of drugs prescribed in the study population (in alphabetical order) and corresponding AzCERT classifications (1 = known risk of QT_c_ prolongation/TdP; 2 = possible risk of QT_c_ prolongation/TdP; 3 = conditional risk of QT_c_ prolongation/TdP; n.l. = not listed)

| **Drug** | **AzCERT classification** | **n** | **%^a^** | |
| --- | --- | --- | --- | --- |
| **All drugs** |  | **857** | **100** | |
| Abiraterone | n.l. | 1 | 0.12 |  |
| Acamprosate | n.l. | 1 | 0.12 |  |
| Acetylsalicylic acid | n.l. | 31 | 3.62 |  |
| Acyclovir | n.l. | 1 | 0.12 |  |
| Agomelatine | n.l. | 1 | 0.12 |  |
| Alendronate | n.l. | 1 | 0.12 |  |
| Allopurinol | n.l. | 7 | 0.82 |  |
| Amiodarone | 1 | 1 | 0.12 |  |
| Amisulpride | 3 | 2 | 0.23 |  |
| Amitriptyline | 3 | 2 | 0.23 |  |
| Amlodipine | n.l. | 26 | 3.03 |  |
| Amoxicillin–clavulanate | n.l. | 1 | 0.12 |  |
| Ampicillin–sulbactam | n.l. | 1 | 0.12 |  |
| Apixaban | n.l. | 9 | 1.05 |  |
| Aripiprazole | 2 | 4 | 0.47 |  |
| Atorvastatin | n.l. | 18 | 2.10 |  |
| Baclofen | n.l. | 1 | 0.12 |  |
| Biperiden | n.l. | 5 | 0.58 |  |
| Bisoprolol | n.l. | 24 | 2.80 |  |
| Bromazepam | n.l. | 1 | 0.12 |  |
| Bupropion | n.l. | 1 | 0.12 |  |
| Calcium | n.l. | 6 | 0.70 |  |
| Candesartan | n.l. | 8 | 0.93 |  |
| Carbamazepine | n.l. | 1 | 0.12 |  |
| Carbimazole | n.l. | 1 | 0.12 |  |
| Cefazolin | n.l. | 1 | 0.12 |  |
| Ceftriaxone | n.l. | 1 | 0.12 |  |
| Chlorprothixene | 1 | 1 | 0.12 |  |
| Ciprofloxacin | 1 | 2 | 0.23 |  |
| Citalopram | 1 | 4 | 0.47 |  |
| Clopidogrel | n.l. | 1 | 0.12 |  |
| Clozapine | 2 | 3 | 0.35 |  |
| Cotrimoxazole | 3 | 1 | 0.12 |  |
| Dalteparin | n.l. | 7 | 0.82 |  |
| Dexamethasone | n.l. | 1 | 0.12 |  |
| Diazepam | n.l. | 9 | 1.05 |  |
| Digoxin | n.l. | 1 | 0.12 |  |
| Dihydralazine | n.l. | 1 | 0.12 |  |
| Donepezil | 1 | 1 | 0.12 |  |
| Doxazosin | n.l. | 1 | 0.12 |  |
| Duloxetine | n.l. | 3 | 0.35 |  |
| Edoxaban | n.l. | 3 | 0.35 |  |
| Enalapril | n.l. | 5 | 0.58 |  |
| Enoxaparin | n.l. | 2 | 0.23 |  |
| Escitalopram | 1 | 3 | 0.35 |  |
| Esomeprazole | 3 | 2 | 0.23 |  |
| Etoricoxib | n.l. | 3 | 0.35 |  |
| Ezetimibe | n.l. | 2 | 0.23 |  |
| Ferrous(II) glycine sulphate complex | n.l. | 1 | 0.12 |  |
| Flucloxacillin | n.l. | 1 | 0.12 |  |
| Flupentixol | 2 | 3 | 0.35 |  |
| Folic acid | n.l. | 8 | 0.93 |  |
| Fresubin® | n.l. | 1 | 0.12 |  |
| Furosemide | 3 | 5 | 0.58 |  |
| Gabapentin | n.l. | 1 | 0.12 |  |
| Glibenclamide | n.l. | 1 | 0.12 |  |
| Glimepiride | n.l. | 1 | 0.12 |  |
| Glycopyrronium bromide | n.l. | 1 | 0.12 |  |
| Haloperidol | 1 | 8 | 0.93 |  |
| Hydrochlorothiazide | 3 | 20 | 2.33 |  |
| Human insulin | n.l. | 2 | 0.23 |  |
| Hydromorphone | n.l. | 1 | 0.12 |  |
| Ibuprofen | n.l. | 4 | 0.47 |  |
| Indacaterol | 3 | 1 | 0.12 |  |
| Insulin glargine | n.l. | 3 | 0.35 |  |
| Irbesartan | n.l. | 1 | 0.12 |  |
| Isosorbide dinitrate | n.l. | 1 | 0.12 |  |
| Lactulose | n.l. | 2 | 0.23 |  |
| Lamotrigine | n.l. | 1 | 0.12 |  |
| Lavender oil | n.l. | 1 | 0.12 |  |
| Lercanidipine | n.l. | 1 | 0.12 |  |
| Levetiracetam | 2 | 6 | 0.70 |  |
| Levodopa–benserazide | n.l. | 5 | 0.58 |  |
| Levothyroxine | n.l. | 30 | 3.50 |  |
| Lisinopril | n.l. | 2 | 0.23 |  |
| Lithium | 2 | 5 | 0.58 |  |
| Lorazepam | n.l. | 36 | 4.20 |  |
| Losartan | n.l. | 2 | 0.23 |  |
| Macrogol 3350 | n.l. | 7 | 0.82 |  |
| Magnesium | n.l. | 1 | 0.12 |  |
| Melperone | 2 | 14 | 1.63 |  |
| Metamizole | n.l. | 18 | 2.10 |  |
| Metformin | n.l. | 12 | 1.40 |  |
| Metoprolol | n.l. | 17 | 1.98 |  |
| Metronidazole | 3 | 1 | 0.12 |  |
| Mirtazapine | 2 | 17 | 1.98 |  |
| Molsidomine | n.l. | 1 | 0.12 |  |
| Morphine | n.l. | 1 | 0.12 |  |
| Methotrexate | n.l. | 1 | 0.12 |  |
| Mucofalk® | n.l. | 1 | 0.12 |  |
| Sodium chloride | n.l. | 2 | 0.23 |  |
| Naloxone | n.l. | 1 | 0.12 |  |
| Nebivolol | n.l. | 2 | 0.23 |  |
| Nitrendipine | n.l. | 1 | 0.12 |  |
| Olanzapine | 3 | 8 | 0.93 |  |
| Omeprazole | 3 | 2 | 0.23 |  |
| Opipramol | n.l. | 2 | 0.23 |  |
| Oxazepam | n.l. | 10 | 1.17 |  |
| Oxycodone | n.l. | 2 | 0.23 |  |
| Pantoprazole | 3 | 39 | 4.55 |  |
| Paracetamol | n.l. | 7 | 0.82 |  |
| Perazine | n.l. | 1 | 0.12 |  |
| Phenoxymethylpenicillin | n.l. | 1 | 0.12 |  |
| Phenprocoumon | n.l. | 4 | 0.47 |  |
| Pipamperone | 2 | 29 | 3.38 |  |
| Potassium chloride | n.l. | 10 | 1.17 |  |
| Prasugrel | n.l. | 1 | 0.12 |  |
| Prednisolone | n.l. | 4 | 0.47 |  |
| Pregabalin | n.l. | 7 | 0.82 |  |
| Promethazine | 2 | 2 | 0.23 |  |
| Propranolol | n.l. | 1 | 0.12 |  |
| Pyridostigmine | n.l. | 1 | 0.12 |  |
| Quetiapine | 3 | 17 | 1.98 |  |
| Ramipril | n.l. | 41 | 4.78 |  |
| Ranitidine | n.l. | 2 | 0.23 |  |
| Rifaximin | n.l. | 1 | 0.12 |  |
| Risperidone | 3 | 36 | 4.20 |  |
| Rivaroxaban | n.l. | 8 | 0.93 |  |
| Sacubitril–valsartan | n.l. | 1 | 0.12 |  |
| Sertraline | 3 | 9 | 1.05 |  |
| Simeticone | n.l. | 1 | 0.12 |  |
| Simvastatin | n.l. | 22 | 2.57 |  |
| Sitagliptin | n.l. | 1 | 0.12 |  |
| Solifenacin | 3 | 2 | 0.23 |  |
| Spironolactone | n.l. | 5 | 0.58 |  |
| Sterofundin® | n.l. | 1 | 0.12 |  |
| Tamoxifen | 2 | 1 | 0.12 |  |
| Tamsulosin | n.l. | 13 | 1.52 |  |
| Thiamazole | n.l. | 1 | 0.12 |  |
| Thiamine | n.l. | 8 | 0.93 |  |
| Tianeptine | n.l. | 1 | 0.12 |  |
| Tilidine–naloxone | n.l. | 4 | 0.47 |  |
| Tinzaparin | n.l. | 3 | 0.35 |  |
| Tiotropium bromide | n.l. | 2 | 0.23 |  |
| Tolperisone | n.l. | 1 | 0.12 |  |
| Torasemide | 3 | 30 | 3.50 |  |
| Tramadol | 2 | 1 | 0.12 |  |
| Tranylcypromine | n.l. | 1 | 0.12 |  |
| Valerian | n.l. | 2 | 0.23 |  |
| Valproate | n.l. | 7 | 0.82 |  |
| Valsartan | n.l. | 5 | 0.58 |  |
| Venlafaxine | 2 | 7 | 0.82 |  |
| Vitamin B complex | n.l. | 7 | 0.82 |  |
| Vitamin B_12_ | n.l. | 2 | 0.23 |  |
| Vitamin D_3_ | n.l. | 12 | 1.40 |  |
| Xipamide | n.l. | 1 | 0.12 |  |
| Zolpidem | n.l. | 1 | 0.12 |  |
| Zopiclone | n.l. | 3 | 0.35 |  |
| Zuclopenthixol | 2 | 1 | 0.12 |  |

^a^Percentages may not total 100 due to rounding.

Abbreviations: AzCERT, Arizona Center for Education and Research on Therapeutics; n.l., not listed; QT_c_, rate-corrected QT; TdP, torsades de pointes.
