# Supplementary material for: Determinants of severe QTc prolongation in a real-world gerontopsychiatric setting
Source: Front Psychiatry. 2023 Mar 23;14:1157996. doi: 10.3389/fpsyt.2023.1157996 (PMC10076587; doi:10.3389/fpsyt.2023.1157996)
Supplement: Supplementary file 2 [file Table_2.docx]

Supplementary Material

**Determinants of QT_c_ prolongation in a real-world gerontopsychiatric setting**

**Martin Schulze Westhoff^1^, Sebastian Schröder^1^, Johannes Heck^2^, Tabea Pfister^1^, Kirsten Jahn^1^, Olaf Krause^3,4^, Felix Wedegärtner^1^, Stefan Bleich^1^, Kai G. Kahl^1^, Tillmann H. C. Krüger^1^, and Adrian Groh^1^**

^1^Department of Psychiatry, Social Psychiatry and Psychotherapy, Hannover Medical School, Hannover, Germany

^2^Institute for Clinical Pharmacology, Hannover Medical School, Hannover, Germany

^3^Institute for General Practice and Palliative Care, Hannover Medical School, Hannover, Germany

^4^Center for Medicine of the Elderly, DIAKOVERE Henriettenstift, Hannover, Germany

*** Correspondence:**Dr. Martin Schulze Westhoff, MD

Department of Psychiatry, Social Psychiatry and Psychotherapy

Hannover Medical School

Carl-Neuberg-Str. 1

30625 Hannover, Germany

Tel.: +49 511 532-7037

Fax: +49 511 532-18523

E-mail: [schulzewesthoff.martin@mh-hannover.de](mailto:schulzewesthoff.martin@mh-hannover.de)

# Supplementary Table

**SUPPLEMENTARY TABLE 2** Frequencies of drug interaction pairs (n = 266) with an association to possible QT_c_ prolongation and AzCERT classifications of involved drugs (1 = known risk of QT_c_ prolongation/TdP; 2 = possible risk of QT_c_ prolongation/TdP; 3 = conditional risk of QT_c_ prolongation/TdP; n.l. = not listed)

| **Drug 1** | **AzCERT classification of drug 1** | **Drug 2** | **AzCERT classification of drug 2** | **n** | |
| --- | --- | --- | --- | --- | --- |
| Pipamperone | 2 | Risperidone | 3 | 10 |  |
| Risperidone | 3 | Torasemide | 3 | 10 |  |
| Pantoprazole | 3 | Risperidone | 3 | 9 |  |
| Mirtazapine | 2 | Pantoprazole | 3 | 8 |  |
| Bisoprolol | n.l. | Risperidone | 3 | 7 |  |
| Melperone | 2 | Risperidone | 3 | 7 |  |
| Mirtazapine | 2 | Pipamperone | 2 | 7 |  |
| Bisoprolol | n.l. | Pipamperone | 2 | 6 |  |
| Pipamperone | 2 | Quetiapine | 3 | 6 |  |
| Pipamperone | 2 | Torasemide | 3 | 6 |  |
| Metoprolol | n.l. | Mirtazapine | 2 | 5 |  |
| Hydrochlorothiazide | 3 | Quetiapine | 3 | 4 |  |
| Hydrochlorothiazide | 3 | Risperidone | 3 | 4 |  |
| Hydrochlorothiazide | 3 | Venlafaxine | 2 | 4 |  |
| Melperone | 2 | Torasemide | 3 | 4 |  |
| Mirtazapine | 2 | Risperidone | 3 | 4 |  |
| Pipamperone | 2 | Sertraline | 3 | 4 |  |
| Bisoprolol | n.l. | Olanzapine | 3 | 3 |  |
| Citalopram | 1 | Pantoprazole | 3 | 3 |  |
| Haloperidol | 1 | Quetiapine | 3 | 3 |  |
| Hydrochlorothiazide | 3 | Olanzapine | 3 | 3 |  |
| Lithium | 2 | Quetiapine | 3 | 3 |  |
| Macrogol | n.l. | Quetiapine | 3 | 3 |  |
| Metoprolol | n.l. | Quetiapine | 3 | 3 |  |
| Mirtazapine | 2 | Quetiapine | 3 | 3 |  |
| Pantoprazole | 3 | Quetiapine | 3 | 3 |  |
| Quetiapine | 3 | Torasemide | 3 | 3 |  |
| Amisulpride | 3 | Pantoprazole | 3 | 2 |  |
| Aripiprazole | 2 | Atorvastatin | n.l. | 2 |  |
| Bisoprolol | n.l. | Melperone | 2 | 2 |  |
| Bisoprolol | n.l. | Mirtazapine | 2 | 2 |  |
| Bisoprolol | n.l. | Quetiapine | 3 | 2 |  |
| Ciprofloxacin | 1 | Pipamperone | 2 | 2 |  |
| Citalopram | 1 | Torasemide | 3 | 2 |  |
| Haloperidol | 1 | Lithium | 2 | 2 |  |
| Haloperidol | 1 | Pipamperone | 2 | 2 |  |
| Haloperidol | 1 | Torasemide | 3 | 2 |  |
| Hydrochlorothiazide | 3 | Melperone | 2 | 2 |  |
| Hydrochlorothiazide | 3 | Sertraline | 3 | 2 |  |
| Metoprolol | n.l. | Venlafaxine | 2 | 2 |  |
| Mirtazapine | 2 | Sertraline | 3 | 2 |  |
| Mirtazapine | 2 | Venlafaxine | 2 | 2 |  |
| Olanzapine | 3 | Pantoprazole | 3 | 2 |  |
| Olanzapine | 3 | Venlafaxine | 2 | 2 |  |
| Pantoprazole | 3 | Sertraline | 3 | 2 |  |
| Prednisolone | n.l. | Risperidone | 3 | 2 |  |
| Risperidone | 3 | Sertraline | 3 | 2 |  |
| Risperidone | 3 | Solifenacin | 3 | 2 |  |
| Sertraline | 3 | Torasemide | 3 | 2 |  |
| Amiodarone | 1 | Torasemide | 3 | 1 |  |
| Amiodarone | 1 | Tramadol | 2 | 1 |  |
| Amisulpride | 3 | Furosemide | 3 | 1 |  |
| Amisulpride | 3 | Opipramol | n.l. | 1 |  |
| Amisulpride | 3 | Promethazine | 2 | 1 |  |
| Amisulpride | 3 | Torasemide | 3 | 1 |  |
| Amitriptyline | 3 | Bisoprolol | n.l. | 1 |  |
| Amitriptyline | 3 | Metoprolol | n.l. | 1 |  |
| Amitriptyline | 3 | Pipamperone | 2 | 1 |  |
| Amitriptyline | 3 | Risperidone | 3 | 1 |  |
| Amitriptyline | 3 | Sertraline | 3 | 1 |  |
| Aripiprazole | 2 | Bisoprolol | n.l. | 1 |  |
| Aripiprazole | 2 | Escitalopram | 1 | 1 |  |
| Aripiprazole | 2 | Pipamperone | 2 | 1 |  |
| Aripiprazole | 2 | Promethazine | 2 | 1 |  |
| Aripiprazole | 2 | Sertraline | 3 | 1 |  |
| Aripiprazole | 2 | Torasemide | 3 | 1 |  |
| Bisoprolol | n.l. | Chlorprothixene | 1 | 1 |  |
| Carbimazole | n.l. | Pipamperone | 2 | 1 |  |
| Ceftriaxone | n.l. | Risperidone | 3 | 1 |  |
| Chlorprothixene | 1 | Haloperidol | 1 | 1 |  |
| Chlorprothixene | 1 | Torasemide | 3 | 1 |  |
| Ciprofloxacin | 1 | Mirtazapine | 2 | 1 |  |
| Ciprofloxacin | 1 | Quetiapine | 3 | 1 |  |
| Ciprofloxacin | 1 | Risperidone | 3 | 1 |  |
| Citalopram | 1 | Digoxin | n.l. | 1 |  |
| Citalopram | 1 | Mirtazapine | 2 | 1 |  |
| Citalopram | 1 | Risperidone | 3 | 1 |  |
| Clozapine | 2 | Escitalopram | 1 | 1 |  |
| Clozapine | 2 | Esomeprazole | 3 | 1 |  |
| Clozapine | 2 | Haloperidol | 1 | 1 |  |
| Clozapine | 2 | Opipramol | n.l. | 1 |  |
| Clozapine | 2 | Pipamperone | 2 | 1 |  |
| Clozapine | 2 | Quetiapine | 3 | 1 |  |
| Clozapine | 2 | Torasemide | 3 | 1 |  |
| Cotrimoxazole | 3 | Quetiapine | 3 | 1 |  |
| Dexamethasone | n.l. | Melperone | 2 | 1 |  |
| Dexamethasone | n.l. | Pantoprazole | 3 | 1 |  |
| Donepezil | 1 | Melperone | 2 | 1 |  |
| Donepezil | 1 | Risperidone | 3 | 1 |  |
| Doxazosin | n.l. | Risperidone | 3 | 1 |  |
| Escitalopram | 1 | Esomeprazole | 3 | 1 |  |
| Escitalopram | 1 | Hydrochlorothiazide | 3 | 1 |  |
| Escitalopram | 1 | Venlafaxine | 2 | 1 |  |
| Flupentixol | 2 | Melperone | 2 | 1 |  |
| Furosemide | 3 | Haloperidol | 1 | 1 |  |
| Furosemide | 3 | Melperone | 2 | 1 |  |
| Furosemide | 3 | Risperidone | 3 | 1 |  |
| Furosemide | 3 | Venlafaxine | 2 | 1 |  |
| Haloperidol | 1 | Olanzapine | 3 | 1 |  |
| Haloperidol | 1 | Perazine | n.l. | 1 |  |
| Lactulose | n.l. | Quetiapine | 3 | 1 |  |
| Lithium | 2 | Olanzapine | 3 | 1 |  |
| Lithium | 2 | Perazine | n.l. | 1 |  |
| Lithium | 2 | Zuclopenthixol | 2 | 1 |  |
| Melperon | 2 | Metoprolol | n.l. | 1 |  |
| Melperon | 2 | Mirtazapine | 2 | 1 |  |
| Melperon | 2 | Pipamperone | 2 | 1 |  |
| Melperon | 2 | Sertraline | 3 | 1 |  |
| Melperon | 2 | Solifenacin | 3 | 1 |  |
| Metoprolol | n.l. | Promethazine | 2 | 1 |  |
| Metoprolol | n.l. | Sertraline | 3 | 1 |  |
| Mirtazapine | 2 | Torasemide | 3 | 1 |  |
| Morphine | n.l. | Risperidone | 3 | 1 |  |
| Nebivolol | n.l. | Risperidone | 3 | 1 |  |
| Olanzapine | 3 | Pipamperone | 2 | 1 |  |
| Olanzapine | 3 | Quetiapine | 3 | 1 |  |
| Olanzapine | 3 | Sertraline | 3 | 1 |  |
| Olanzapine | 3 | Torasemide | 3 | 1 |  |
| Opipramol | n.l. | Promethazine | 2 | 1 |  |
| Opipramol | n.l. | Torasemide | 3 | 1 |  |
| Oxycodone | n.l. | Pipamperone | 2 | 1 |  |
| Pantoprazole | 3 | Tramadol | 2 | 1 |  |
| Pipamperone | 2 | Prednisolone | n.l. | 1 |  |
| Pipamperone | 2 | Propranolol | n.l. | 1 |  |
| Pipamperone | 2 | Venlafaxine | 2 | 1 |  |
| Prednisolone | n.l. | Sertraline | 3 | 1 |  |
| Promethazine | 2 | Sertraline | 3 | 1 |  |
| Promethazine | 2 | Tamoxifen | 2 | 1 |  |
| Quetiapine | 3 | Venlafaxine | 2 | 1 |  |
| Quetiapine | 3 | Zuclopenthixol | 2 | 1 |  |
| Risperidone | 3 | Melperone | 2 | 1 |  |
| Risperidone | 3 | Pipamperone | 2 | 1 |  |
| Risperidone | 3 | Sitagliptin | n.l. | 1 |  |
| Torasemide | 3 | Quetiapine | 3 | 1 |  |
| Torasemide | 3 | Tramadol | 2 | 1 |  |
| Torasemide | 3 | Zuclopenthixol | 2 | 1 |  |

Abbreviations: AzCERT, Arizona Center for Education and Research on Therapeutics; n.l., not listed; QT_c_, rate-corrected QT; TdP, torsades de pointes.
